# Prevalence of HIV in Medicare beneficiaries with lung cancer

**DOI:** 10.1186/1750-9378-7-S1-P21

**Published:** 2012-04-19

**Authors:** Jeannette Y Lee, Page C Moore

**Affiliations:** 1 Department of Biostatistics, University of Arkansas for Medical Sciences, Little Rock, AR, USA

## Background

Human immunodeficiency virus (HIV) patients are at higher risk for lung cancer than the general population [1]. The impact of HIV on the lung cancer population is unclear. In this study, we estimate the prevalence of HIV among Medicare beneficiaries diagnosed with lung cancer.

## Material and methods

This study used the SEER-Medicare database which links Medicare claims data with patients identified through cancer registries as part of the Surveillance Epidemiology and End Results (SEER) program. There were 250,500 patients who were diagnosed with malignant lung cancer between 1998 and 2007: 225,233 qualified for Medicare based on age and were 65 years or older at diagnosis and 25,267 qualified for Medicare based on disability and were less than 65 years old at diagnosis. Demographic information was taken at the time of the initial lung cancer diagnosis. Patients were classified as prevalent HIV cases if their first Medicare claim with a diagnosis of HIV preceded the diagnosis of lung cancer or occurred within one year after lung cancer was diagnosed. Relative risk (RR) was used to assess risk factors.

## Results

The prevalence of HIV in lung cancer cases was 180.6 (95% CI: 163.8 to 199.0) and 1,646.0 (95% CI: 1,495.1 to 1,812.3) per 100,000 among elderly and disabled beneficiaries, respectively. It doubled from 1998 to 2007 for elderly beneficiaries and increased by 35% for disabled beneficiaries. Risk factors for HIV were male gender, non-white race, never having been married, and residence in a metropolitan area (Table 1).

**Table 1:** 

	Elderly Beneficiaries RR	Disabled Beneficiaries RR
Male vs. Female	1.9*	3.5*
African-American vs. White	6.4*	3.1*
Other race vs. White	2.0*	0.7
Never married vs. Other (Men)	4.8*	7.3*
Never married vs. Other (Women)	2.2*	4.6*
Big Metro vs Other	5.5*	5.2*
Metro vs. Other	2.9*	2.9*

*RR significantly > 1.0

Elderly HIV and non-HIV patients were comparable with respect to stage of lung cancer at diagnosis, but HIV-infected disabled beneficiaries were more likely to present with distant metastases than their non-HIV counterparts.

## Conclusions

The prevalence of HIV among elderly Medicare beneficiaries with lung cancer was 2.6 higher than in the general population 65 years and older [2]. For disabled beneficiaries, the prevalence of HIV among lung cancer cases was higher than for those without lung cancer [3]. The increasing prevalence of HIV in lung cancer cases may result in a commensurate increase in demand for health care services for Medicare beneficiaries.
